# Short-term and long-term effectiveness of acupuncture and Tuina on knee osteoarthritis: study protocol for a randomized controlled trial

**DOI:** 10.3389/fneur.2023.1301217

**Published:** 2023-11-24

**Authors:** Rui-li Zhao, Pei-hong Ma, Bao-yan Liu, Chang-he Yu, Hao-ran Zhang, Qian Lv, Da-wei Yang, Yu-ping Yang, Hong-yan Liu, Fu-yu Wang, Chun-sheng Yin, Shao-guang Su, Hong-chi Wang, Xi-you Wang, Shi-yan Yan

**Affiliations:** ^1^Acupuncture and Moxibustion Department, Beijing University of Chinese Medicine, Beijing, China; ^2^China Academy of Chinese Medical Sciences, Beijing, China; ^3^Dongzhimen Hospital of Beijing University of Chinese Medicine, Beijing, China; ^4^College of Preschool Education, Beijing Youth Politics College, Beijing, China; ^5^Guang'anmen Hospital (Southern District), China Academy of Chinese Medical Sciences, Beijing, China; ^6^Weifang Hospital of Traditional Chinese Medicine, Weifang, China; ^7^Shunyi Hospital of Beijing Traditional Chinese Medicine Hospital, Beijing, China

**Keywords:** knee osteoarthritis, acupuncture, tuina, exercise, randomized controlled trial, telehealth

## Abstract

**Background:**

The effectiveness of acupuncture and tuina in treating knee osteoarthritis (KOA) is still controversial, which limits their clinical application in practice. This study aims to evaluate the short-term and long-term effectiveness of acupuncture and tuina on KOA.

**Methods/design:**

This parallel-group, multicenter randomized clinical trial (RCT) will be conducted at the outpatient clinic of five traditional Chinese medicine hospitals in China. Three hundred and thirty participants with KOA will be randomly assigned to acupuncture, tuina, or home-based exercise group with a ratio of 1:1:1. The primary outcome is the proportion of participants achieving a minimal clinically important improvement defined as a ≥ 12% reduction on the Western Ontario and McMaster Universities Osteoarthritis Index (WOMAC) pain dimension on short term (week 8) and long term (week 26) compared with baseline. Secondary outcomes are knee joint conditions (pain, function, and stiffness), self-efficacy of arthritis, quality of life, and psychological conditions, which will be evaluated by the WOMAC score and the Patient Global Assessment (PGA), and in addition, the respondents index of OMERACT-OARSI, Short Form 12 Health Survey (SF-12), arthritis self-efficacy scale, and European five-dimensional health scale (EQ-5D). Adverse events will be collected by self-reported questionnaires predefined.

**Clinical trial registration:**

https://www.chictr.org.cn

## 1 Introduction

Knee osteoarthritis (KOA) is the most common type of osteoarthritis, characterized by chronic pain and impaired activity function that significantly impacts the activities of daily living and quality of life of patients (1). In 2019, the global age-standardized prevalence rate (ASR) of OA was 6348.25 per 100,000, with KOA accounting for approximately 60.6% (2). In China, the number of prevalent cases of KOA has increased from approximately 110 million in 2016 (3) to 132.81 million in 2019 (2), with 75% of patients aged 45–74 years (2). From a public health point of view, KOA imposes an increasing economic burden on individuals and society (4).

Acupuncture is increasingly being used as a complementary alternative therapy during medical interventions, and substantial patients are willing to use it for disease treatment and prevention (5, 6). Analgesia is one of the primary effects of this therapy (7). Although studies have found that acupuncture benefits function, pain, and knee stiffness (8–10), its effectiveness in treating KOA is still equivocal (11), and most studies have only focused on its short-term benefits. A meta-analysis showed that acupuncture can alleviate pain and improve function in both the short term and long-term (12) but more rigorously designed studies are needed to investigate its medium-term or long-term effectiveness (13, 14). As another non-invasive and non-pharmaceutical therapy (15), tuina is widely accepted as a medical intervention in Asia. Some previous studies have shown that it is effective in relieving pain and improving function for some musculoskeletal diseases such as neck pain, back pain, and KOA (16). However, evidence of its effectiveness on KOA is lacking, and well-designed studies estimating its medium-term and long-term effectiveness are rare so far (16). Thus, considering the chronic and degenerative characteristics of KOA (17), it is of utmost importance to assess the long-term effectiveness of acupuncture and tuina for patients with KOA. Additionally, exercise programs are currently adopted among KOA patients and predisposing effectiveness has been observed (18, 19). Consistent with this, most guidelines recommended health education and management, weight loss, and exercise therapy as the core treatments for KOA (20, 21). However, the application of exercise and physicians capable of directing appropriate exercise therapy are limited (22, 23), and continuous practitioner involvement may be impractical due to cost and/or work time. Based on the increasing popularity of mobile video in daily life, it is possible to design a convenient platform to support online exercise programs to guide patients’ exercise.

Therefore, the primary aim of our randomized controlled trial (RCT) is to assess the short-term and long-term effectiveness of acupuncture or tuina, respectively, compared with home-based exercise with an online program in treating KOA.

## 2 Methods

### 2.1 Study design and setting

This is a parallel multicenter, pragmatic, randomized controlled study that will be performed at five centers in China between October 2021 and December 2025. These centers were selected as they are representative of TCM of these hospitals and the distribution of patients with KOA in the local communities: (1) Dongzhimen Hospital of Beijing University of Chinese Medicine, (2) Guang’anmen Hospital (Southern District), China Academy of Chinese Medical Sciences, (3) Shunyi Hospital of Beijing Traditional Chinese Medicine Hospital, (4) Weifang Hospital of Traditional Chinese Medicine, (5) The Affiliated TCM Hospital of Guangzhou Medical University.

**Figure 1:**
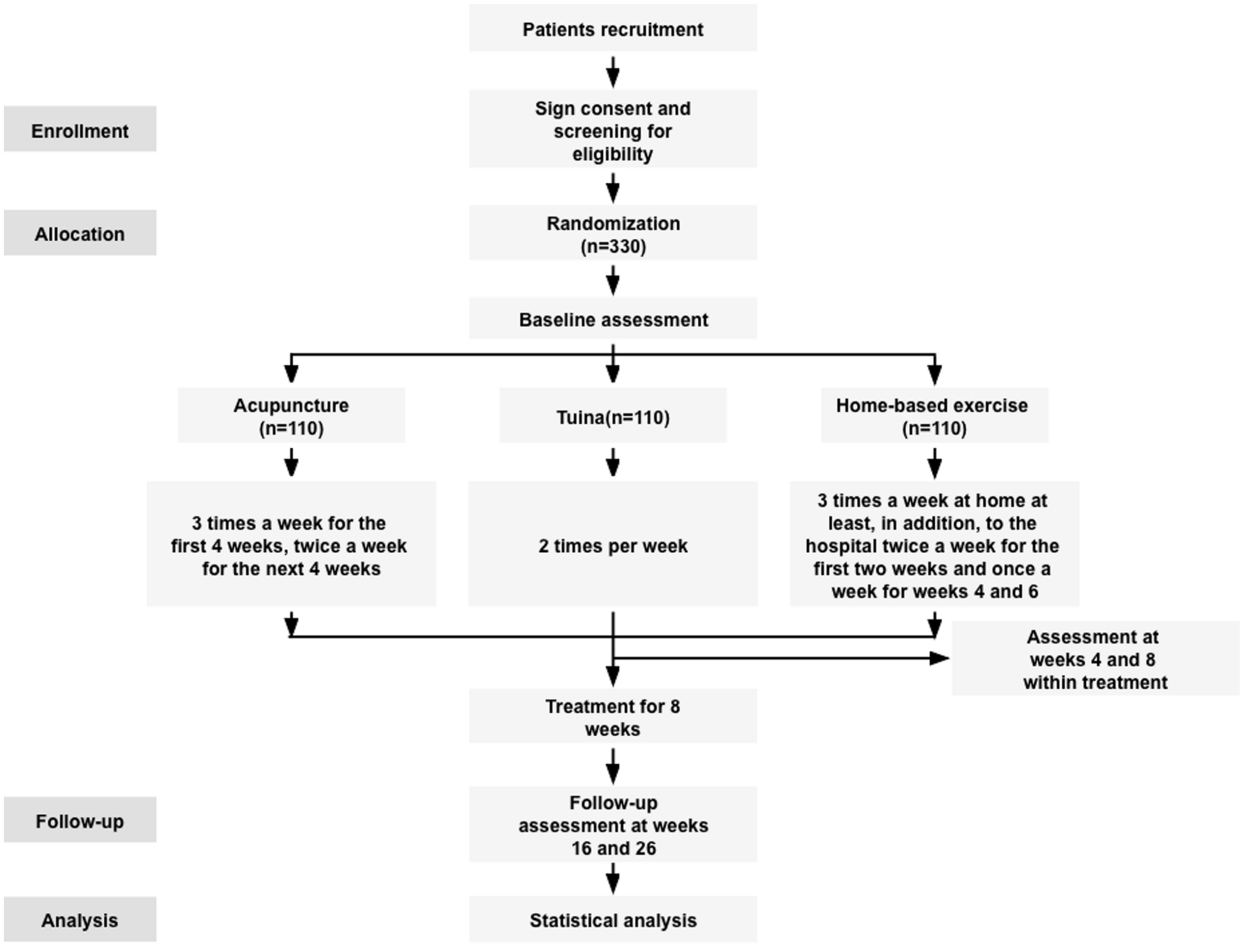
Flow diagram of the study participants enrollment and follow-up.

The trial has been approved by the Ethics Committee of Beijing University of Chinese Medicine (2023BZYLL0708), completing the ethical process per sub-center, and it has been registered on the Chinese Clinical Trial Registry (ChiCTR2200058089). This study will report following the guidelines of SPIRIT (24). Figure 1 shows the flowchart of the study design.

### 2.2 Participants

The diagnosis (1. of Supplementary material) criteria of KOA was made based on the criteria of the Chinese Orthopaedic Association (25) and the American College of Rheumatology (26). Eligible participants should: (1) be aged 45–75 years; (2) Kellgren–Lawrence (KL) grade (27) II or III; (3) have average knee pain over 1 week of the numerical rating scale (NRS) ≥ 4; (4) be suffered from pain in the last 3 months; (5) sign the informed consent form. The exclusion criteria are as follows: (1) knee pain caused by other diseases (such as infectious arthritis) or comorbid contusion or other trauma; foot deformity, pain, and other diseases affecting walking; serious osteoporosis; the skin around the knee joint broken or suffered from dermatosis that affect manipulation; (2) history of knee surgery or waiting for knee surgery; arthroscopy within 12 months; intra-articular injection within 6 months; medication for treating KOA within 1 week; (3) history of knee exercise program within 6 months or comorbidities that affect lower limb motor ability, balance ability, or strength training, such as stroke, myocardial infarction, peripheral neuropathy, Parkinson’s disease, and multiple sclerosis; (4) history of receiving acupuncture or tuina therapy within 3 months or participating in another clinical study in the previous 3 months; (5) history of fainting during acupuncture; (6) comorbidities such as serious cardiovascular and cerebrovascular diseases, mental disorders, malignant tumors, coagulation disorders, liver and kidney dysfunction, and severe gastrointestinal diseases; (7) pregnant or breastfeeding or with a pregnancy plan; (8) not familiar with using smartphones. Participants who meet any of the above exclusion criteria will be excluded.

### 2.3 Sample size

According to the previous studies (28), the baseline value of the WOMAC pain subscale in Chinese KOA patients was obtained as a mean difference of 6.5 (SD = 2.8) on the Likert scale, scored on a range of 0–4 points. Using which the pain subscale value was transformed to 16.25 (standard deviation [SD] = 7) on the 0–10 points NRS scale. The Minimum Clinically Important Difference (MCID) of the WOMAC pain subscale is a reduction of at least 12% (29). We estimate that there will be a difference of at least 12% between acupuncture and home-based exercise groups, or tuina and home-based exercise groups, in the WOMAC pain subscale after 8 weeks of treatment. Furthermore, we anticipate this difference to be maintained at the 26-week follow-up, with a value of 1.95 (SD = 7). Using a Bonferroni correction method to avoid the inflation of type I errors, a two-sided significance level of 0.025 was set for two independent test hypotheses, namely, acupuncture vs. home-based exercise and tuina vs. home-based exercise. To achieving a power of 80%, 82 participants will be needed in each group. Assuming a drop-out rate of 25%, a total of 330 eligible participants with 110 participants per group will be needed.

### 2.4 Recruitment, randomization, and blinding

Participants will be recruited from the outpatient departments at each center as well as through various strategies that involve the use of a hospital information system (HIS). These methods may include posting posters in the clinics and distributing e-posters on social media platforms, such as WeChat.

Prior to any study procedures, potential participants will be recruited sequentially from each center. Written informed consent will be obtained from potential participants during the screening and eligibility assessment phase. Those who meet the eligibility criteria will be randomly assigned in a 1:1:1 ratio to either acupuncture or tuina or home-based exercise groups, stratified by a study center. The randomization sequence will be generated using the PROC PLAN process in SAS (version 9.3). The central randomization system developed by the Institute of Basic Research in Clinical Medicine at the China Academy of Chinese Medical Sciences will implement the group assignment. An independent statistician, who will not conduct the evaluation or statistical analysis of the trial, will ensure adequate randomization.

While the participants and physicians will not be blinded to interventions, the outcome evaluators and biostatisticians will be blinded to group details.

### 2.5 Interventions

**Figure 2:**
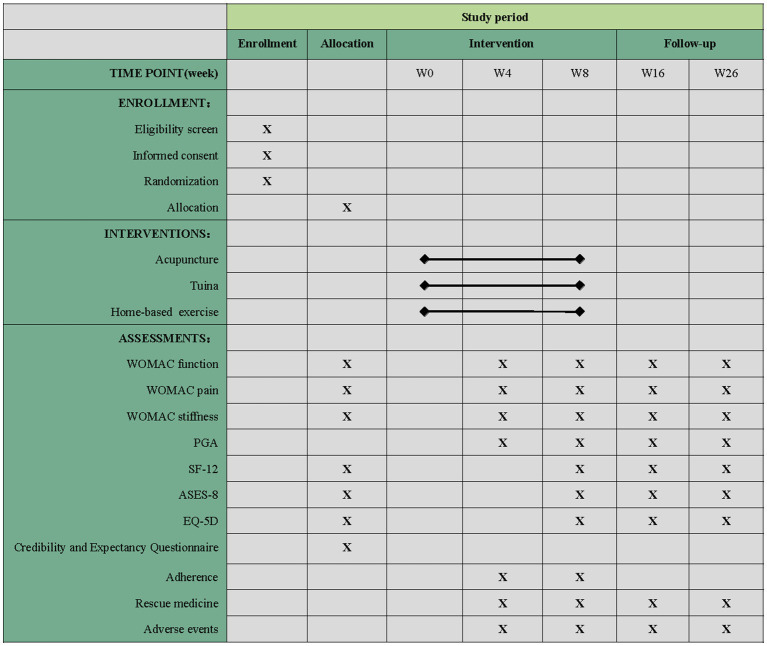
Schedule of enrollment, intervention, and assessments of this study protocol. WOMAC, Western Ontario and McMaster Universities Osteoarthritis Index; PGA, Patient Global Assessment; SF-12, 12-item Short Form Health Survey; ASES, Arthritis Self Efficacy Scale; EQ-5D, European Five-Dimensional Health Scale. “X” projects that must be completed.

Each participant will receive a usual care education package for KOA, excluding the corresponding treatment. This package will provide basic knowledge of osteoarthritis, pain management, treatment recommendations, physical activity administration, the benefits of exercise, and health notes for KOA. Our study team has integrated these educational materials into a mobile phone application (APP) called *Knee for long*, which is specially designed for patients with KOA by our team. The APP includes various modules for health records, self-management, and clinical research. Through the APP, participants will receive the same educational information via tweets, recordings, and videos regarding the causes, symptoms, and main treatment measures of KOA. To ensure consistent treatment time across all three groups, practitioners will only treat the worse knee if participants with bilateral KOA. The schedule of enrollment, interventions, assessments, and participant visits are shown in Figure 2.

#### 2.5.1 Acupuncture group

**Figure 3:**
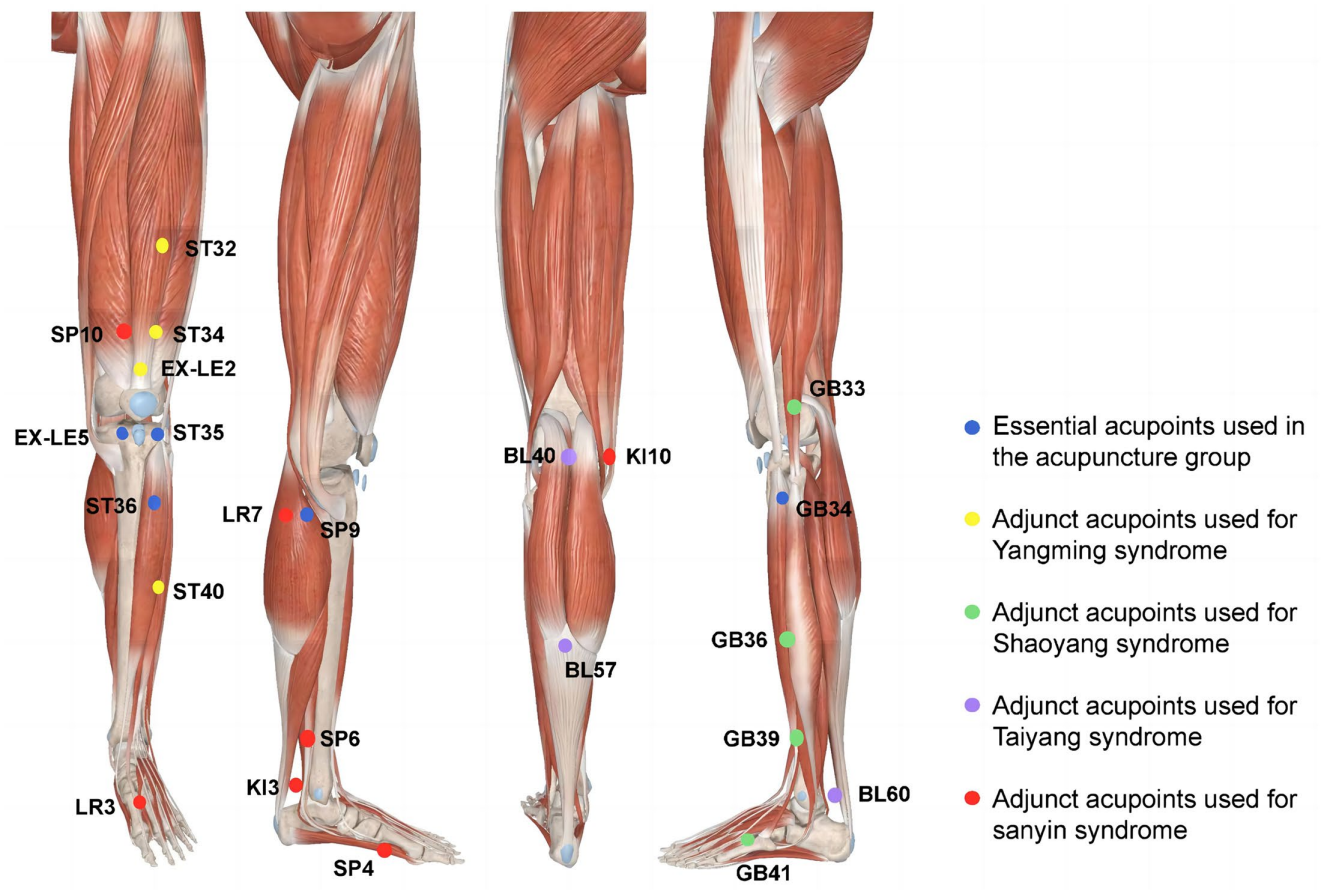
Locations of acupoints. Blue dots: essential acupoints used in the acupuncture group. Yellow dots: adjunct acupoints used for YangMing syndrome. Green dots: Adjunct acupoints used for ShaoYang syndrome. Purple dots: Adjunct acupoints used for TaiYang syndrome. Red dots: Adjunct acupoints used for three-yin syndrome (Note: Modified based on 3D body).

The acupuncture prescription was developed based on previous clinical research (8) and expert consensus (2.0 of Supplementary material and Figure 3). It is semi-standardized, incorporating six essential acupoints and three adjunct acupoints. The essential acupoints are *Dubi* (ST35), *Neixiyan* (EX-LE5), *Zusanli* (ST36), *Yinlingquan* (SP9), *Yanglingquan* (GB34), and an *Ashi* point (where the participant feels the worst pain). Selection of the adjunct acupoints will be based on the location of pain according to meridians. All acupoints will be localized according to the Nomenclature and Location of Acupuncture Points in the People’s Republic of China in 2021 (GB/T 12346–2021) (30) (2.0 of Supplementary material). If the pain is located in the anterior aspect of the affected knee joint, three YangMing acupoints will be chosen from *Futu* (ST32), *Liangqiu* (ST34), *Heding* (EX-LE2), and *Fenglong* (ST40). For lateral pain, indicative of ShaoYang syndrome, three adjunct acupoints will be selected from *Waiqiu* (GB36), *Xuanzhong* (GB39), *Zulinqi* (GB41), and *Xiyangguan* (GB33). For posterior pain, associated with TaiYang syndrome, adjunct acupoints will be *Weizhong* (BL40), *Chengshan* (BL57), and *Kunlun* (BL60). For medial pain, indicative of three-yin syndrome, three adjunct acupoints will be chosen from *Xuehai* (SP10), *Yingu* (KI10), *Xiguan* (LR7), *Sanyinjiao* (SP6), *Taixi* (KI3), *Taichong* (LR3), and *Gongsun* (SP4). If more than two meridians are affected, three relevant adjunct acupoints will be selected from those for the corresponding syndromes.

The treatment will be performed by licensed acupuncturists or tuina practitioners who have a minimum of 5 years of experience in treating KOA. Prior to the study, they will be trained in the implementation of standardized operating procedures (SOP) at each center. To ensure consistency, a detailed instruction manual and video will be provided to them.

Uniform disposable sterile filiform needles (0.25 mm in diameter, 40–50 mm in depth) from Tianjin Yipeng Medical Device Co., Ltd. in China will be used in each center. After skin disinfection, acupuncturists will insert needles into the predetermined acupoints to induce the deqi sensation, characterized by soreness, numbness, distention, and heaviness. During the 30 min retention, a small amplitude of uniform lifting and thrusting twirling lasting 10–15 s will be performed every 10 min to sustain the deqi sensation. The acupuncture treatment will consist of 20 sessions over 8 weeks (3 sessions per week for the first 4 weeks, 2 sessions per week for the subsequent 4 weeks).

#### 2.5.2 Tuina group

Tuina therapy consists of six standardized steps based on the expert nation-wide consensus on the manipulation of tuina for KOA (31) and clinical experts’ suggestions. The procedure will be precisely defined in terms of actions, duration, frequency, and intensity to ensure consistency (Table 1). The manipulation intensity should be tolerated to the participant’s tolerance and violent manipulation will be prohibited. The tuina therapy will comprise 16 sessions of 25 min in duration, held over 8 weeks with two sessions per week.

**Table 1:** Detailed manipulation of tuina.

Relaxation	Prone position: relaxing the bladder meridian of foot-TaiYang (from the hip’s horizontal grain to the Achilles tendon) on the affected limb’s back. Performing rolling manipulation, poking manipulation, and pushing manipulation with palm root, respectively, for five times, a total of 3.5 min.	
Press the acupoints	Supine position: acupressure will be applied to specific acupoints: *Xuehai* (SP10) - *Liangqiu* (ST34), *Dubi* (ST35) *Neixiyan* (EX-LE5), *Zusanli* (ST36), *Yinlingquan* (SP9), *Yanglingquan* (GB34) will be pressed using the belly of the thumb and index finger, with gradual and continuous from light to heavy. Each acupoint pair will be pressed for 30 s, a total of five times.	
	The medial and lateral collateral ligaments will be plucked with the thumb or four fingers for five times, approximately 1 min.	
Adjust muscles and tendons	Plucking the head of the gastrocnemius muscle and the hamstring muscle from the top to the bottom using the four-finger pulp of both hands back and forth for five times, approximately 1 min.	
Settle patella	Grabbing patella	Pushing the patella to the medial side with one thumb, and slowly grabbing the patella from bottom to top along the inner edge of the patella with the other four fingers. This action will be repeated once, with two hands and the direction reversed, approximately 1 min.
	Kneading patella	Kneading on the affected patella by using the palm with clockwise or counterclockwise direction, for 2 min.
Move knee joint	Flexion-extension-distraction manipulation of the knee	Supine position: the hip and knee joints will be flexed at a 90° angle. The four fingers will hold the lower part of the knee joint in the popliteal fossa while the two thumbs face upwards. Then swinging up and down the knee joint for 10 times and stretched out once. The procedure will be repeated five times, approximately 2 min.
	Hamstring stretch	Making the ankle dorsiflexed with one hand using a steady force, while the other hand is placed on the knee joint. The ankle will be extended as far as the participant can tolerate. The operation will be held for 10–15 s with five times, approximately 1 min.
Ending manipulation	Pushing the stomach meridian of foot-YangMing, using the palm root or thenar apply pressure on the front thigh, for 10 times, approximately 1 min.	
After the manipulation, lying down and resting for 3 min.		

#### 2.5.3 Home-based exercise group

The home-based exercise program, based on previous clinical research and self-management mode (19, 32), will be incorporated into the APP. Participants will receive the same educational materials as the other two groups, along with an online home-based stepped exercise program accessible through the APP. Only participants allocated to the home-based exercise group will have access to the exercise module, which contains instructions, prerecorded exercise videos, time consumption, and assessments of difficulty level. During the first 2 weeks, participants will be required to receive guidance on exercise in hospitals twice a week, while in the 4th and 6th weeks once a week, there is a need to assess and guide the accuracy of their movements. Based on guidance in hospital, participants will be informed to carry out the exercise program at home at least three times per week according to the videos provided. Each training and the duration of the exercise will be recorded by the APP. The exercise program includes three parts: preparation activity, formal exercise program (comprising five subsections), and the ending stage. Each program has various subsections, and the formal exercise program will also have three intensity levels. The APP will distribute different levels to participants based on their post-exercise evaluations. If any subsection evaluated fulfills the easy level (three levels: easy, moderate, and difficult), the exercise intensity level will be increased by adding ankle weights and/or changing body position. If the entire program is evaluated as moderate in level four or less for at least three days on a modified Borg Rating of Perceived Exertion scale (33), the next stage will be unlocked. Additional detailed exercise sessions and videos can be found in 3.0 of Supplementary material, providing detailed instructions for the program.

During the trial, other therapies and any analgesic medications relevant to KOA will be prohibited. All participants will be provided with acetaminophen (tylenol) after enrollment. If their symptoms worsen and become unbearable, participants may take acetaminophen (325 mg.qd, with a maximum dose of 2,600 mg.qd). Each use of acetaminophen should be recorded promptly. Participants should be questioned about any other medications for other diseases in detail during the first and last two visits.

### 2.6 Outcomes

The efficacy of the intervention will be evaluated based on knee joint conditions (pain, function, and stiffness), self-efficacy of arthritis, quality of life, and psychological conditions. Endpoints will be recorded and assessed on the 8th week and 26th week after initial treatment.

#### 2.6.1 Primary outcomes

The primary outcomes of the intervention will be measured by the improvement of the Western Ontario and McMaster Universities Osteoarthritis Index (WOMAC) pain subscale after 8 and 26 weeks as compared with baseline levels. The WOMAC (34) is a disease-specific measure used to evaluate pain, stiffness, and joint function for KOA. Each of the 24 items is rated on a 0–10 numeric rating scale, with total scores ranging from 0 to 240 points. The subscales consist of 5, 2, and 17 items for pain, stiffness, and functional assessment, respectively. Lower scores indicate a milder symptom for each subscale.

#### 2.6.2 Secondary outcomes

##### 2.6.2.1 WOMAC

Changes in total scores, stiffness, and function subscales of WOMAC will be assessed at the baseline and weeks 4, 8, 16, and 26. The WOMAC pain subscale will be assessed at the baseline and weeks 4 and 16.

##### 2.6.2.2 Patient global assessment

This scale will assess the overall degree of improvement at weeks 4, 8, 16, and 26 by inquiring how participants feel about their knee. PGA consists of seven options: (1) very much improved, (2) much improved, (3) minimally improved, (4) no change, (5) minimally worse, (6) much worse, and (7) very much worse (35).

##### 2.6.2.3 The respondents index of OMERACT-OARSI

This study measures clinical outcomes for osteoarthritis, co-developed by Osteoarthritis Research Society International (OARSI) and the Outcome Measures in Rheumatology (OMERACT). OMERACT-OARSI response criteria will be calculated based on pain and functional subscales of WOMAC and PGA at weeks 4, 8, 16, and 26. The pain and function subscales of WOMAC scores will be converted to a 0–100 point scale by multiplying (100/50) and (100/170), respectively. The PGA score will be multiplied by 10 and converted to a 0–100 point scale. Scores meeting the following criteria will be considered clinically relevant: (1) improvement of ≥50% in WOMAC pain or function subscales and an absolute change of ≥20 points; (2) improvement of ≥20% in WOMAC pain or function subscales and an absolute change of ≥10 points or PGA improvement of ≥20% with an absolute change of ≥10 points.

The following scales will be assessed at weeks 8, 16, and 26 (36).

##### 2.6.2.4 Short form 12 health survey

SF-12 is a tool that measures the quality of life in both physical and mental health, using a 12-item questionnaire that evaluates eight dimensions: general health, physical functioning, role physical, bodily pain and energy, social functioning, role emotional, and mental health. Higher scores on the questionnaire correspond to a better quality of life (37).

##### 2.6.2.5 Arthritis self-efficacy scale

The ASES-8 scale is a shorter version of the tool that measures three dimensions of pain, function, and other symptoms in just eight items. Each item is scored from 1 to 10, and the final score is the mean of the 8 items. A higher score indicates a stronger sense of self-efficacy (38).

##### 2.6.2.6 European five-dimensional health scale

This scale consists of the EQ-5D-5L and the EQ-VAS. The EQ-5D-5L evaluates five dimensions: mobility, self-care, usual activities, pain/discomfort, and anxiety/depression. Each dimension corresponds to a question, with five answer levels. The EQ-VAS is a vertical visual scale that ranges from 0 (worst) to 100 (best) health. The utility value scoring system converts the results into a final quality of life (39).

##### 2.6.2.7 Proportion of rescue medication

During each visit, participants will be asked if they have used emergency medications in order to evaluate the proportion of taking emergency medication during the study period.

##### 2.6.2.8 Credibility and expectancy questionnaire

The efficacy expectation scale will only be evaluated at baseline (40).

#### 2.6.3 Adverse events

Any adverse events and related information, such as occurrence time, symptoms, severity, and duration will be inquired and recorded at weeks 4, 8, 16, and 26 by the outcome assessor. Specific adverse events related to acupuncture, tuina, and home-based exercise will be predefined due to the unique characteristics of each intervention. Acupuncture-related adverse events include unbearable acupuncture pain, severe pain lasting more than 1 h (VAS ≥ 4 on a 10-point scale), local hematoma, metal allergy, bent, stuck, or broken needle, fainting, pneumothorax, nerve injury, visceral injury, and others. Tuina-related adverse events include swelling of the treated area, subcutaneous damage, subcutaneous bleeding, delayed onset muscle soreness (DOMS) (41), fracture, and others. Adverse events associated with home-based exercise include falls, sprain, muscle and ligament strains, DOMS, and others. Generic adverse events that occur during the study will also be recorded.

### 2.7 Data management

We will apply an Electronic Data Capture system to collect the data (eCRF). Independent evaluators at each site, who are not involved in allocation or treatment, will enter the data generated in this trial. The database will be locked upon completion, preventing modifications by researchers. Any changes needed in the data must be approved by the project leader and documented by the data management unit. Both paper and electronic documents will be retained for 5 years after publication. To ensure participant confidentiality and prevent information leakage, participant information will remain anonymous.

### 2.8 Quality control

In the process of design, the expert in acupuncture, orthopedics, methodology, and statistics demonstration meeting will be conducted to guarantee the scientificity and rationale of the trial design. A pre-specified SOP will be used in each procedure. Online monitoring and on-site monitoring will be applied in the study. All modifications of the data and the reasons for modifications can be found through the eCRF.

### 2.9 Statistical analysis

The intention-to-treat (ITT) analysis including all randomized participants from the three groups will be used. The primary outcomes will be analyzed using a mixed-effects model of repeated measures to compare the changes in the WOMAC pain subscale between the acupuncture or tuina group and the home-based exercise group at weeks 8 and 26 as compared to baseline. Similarly, changes in the WOMAC function subscale and stiffness subscale will also be analyzed using the same methodology. The response rate of OMERACT-OARSI will be assessed using the chi-square test. For SF-12 and ASES-8, analysis of covariance will be employed to evaluate differences in the total score improvements between groups compared to baseline. Differences in the PGA between groups will be evaluated using the Kruskal–Wallis test. Multiple imputations will be used for missing data. The chi-square or Fisher’s exact test will be used to compare the incidence of adverse events.

## 3 Discussion

This study aims to assess the short-term and long-term effectiveness of acupuncture and tuina in treating KOA as compared to home-based exercise using telehealth programs. This study will prompt to lay a more evidential foundation for the selection of KOA treatments.

Acupuncture and tuina are among the most widely used traditional Chinese medicine (TCM) therapies for treating KOA in clinical practices (42, 43). However, there is a relative lack of supportive high-quality evidence in the literature, and only limited studies with low quality have shown that acupuncture and tuina were effective in reducing pain and improving function in individuals with chronic degenerative osteoarthritis (44, 45). Meanwhile, these studies have primarily focused on the immediate and short-term effectiveness of acupuncture and tuina (12, 46), and the evidence regarding long-term outcomes is equivocal. For example, studies comparing acupuncture versus sham acupuncture for KOA have shown inconsistent results after 26 weeks of treatment (46). Therefore, this study is designed to further explore the effectiveness of acupuncture and tuina for both short-term and long-term outcomes in a well-designed, large-scale trial. Self-management with physical exercise will serve as the standard control, as recommended in current various international guidelines (21, 22, 47).

An 8-week acupuncture prescription is adapted based on a previous study conducted by our team (8), which is a combination of adjacent, local, and distant points to achieve acupuncture analgesia and promote knee function recovery (48, 49). While the previous trial validated the regimen’s short-term effectiveness, a rigorous RCT is needed to establish its long-term effectiveness. For our tuina regimen, we mainly adapted the Institution Standardized Regimen of Tuina for KOA, which is based on an expert consensus (31). The regimen aims to eliminate neutrophils caused by the injured muscle tissue and inhibit the release of inflammatory factors, thereby relieving pain, promoting muscle recovery (50), and relieving local or even lower limb dysfunction (51, 52). Prior to the implementation of the sub-center, we trained all the researchers to ensure the consistency of every operation. To guarantee the quality of the study, only those who passed the SOP examination could be eligible to conduct the trial.

We designed a stepped-exercise program for our study, which is highly beneficial for participants’ self-management. These videos focus on strength and function training of lower limbs to improve core strength, adjust the structural mechanics of the knee joint, and reduce pain (53). To optimize acceptability, we have designed prerecorded exercise videos to vary in difficulty, allowing participants to choose their intensity level and progress at their own pace. The videos will be integrated into the APP for the home-based group, providing participants with the flexibility to customize their exercise intensity according to their individual tolerance level. Besides providing exercise guidance, this APP also offers a reminder function to enhance adherence. To enhance the degree of completion and exercise accuracy of participants in the home-based exercise group, they will be invited to our sub-center for face-to-face guidance twice a week for the first two weeks and once a week for week 4 and week 6.

Our study has a few limitations. First, considering the distinct intervention characteristics of acupuncture, tuina, and home-based exercise, blinding participants and physicians is impossible. However, we will take the following measures so as to avoid the crossover between the three groups. We will train all the physicians before implementing the trial of the first participant, and the physicians for each group will be fixed and treated independently. For participants, it is not allowed to change groups after randomization. If a participant changes group, he/she will discontinue the study, and just the data before group changing will be analyzed. Nevertheless, a study demonstrated that blinding may be of lower importance than is commonly believed in randomized controlled trials (54). To minimize the risk of potential bias, evaluators and statisticians were blinded to the group assignments for this study. Secondly, since we only enrolled patients with mild-to-moderate level KOA symptoms, the effectiveness of severe symptoms might not be observed in our study, thereby potentially limiting the generalization of the findings.

To summarize, this study is expected to produce valuable evidence regarding the short-term and long-term effectiveness of acupuncture or tuina as complementary alternative therapies for KOA. Meanwhile, the findings of this study may help to enhance the clinical application of these therapies in the treatment of KOA.

## Ethics statement

The studies involving humans were approved by the Clinical Research Ethics Committee of Beijing University of Chinese Medicine. The studies were conducted in accordance with the local legislation and institutional requirements. The participants provided their written informed consent to participate in this study.

## Author contributions

R-lZ: Writing - original draft, Project administration. P-hM: Writing - original draft, Project administration. B-yL: Methodology. C-hY: Methodology. H-rZ: Data curation. QL: Investigation. D-wY: Investigation. Y-pY: Investigation. H-yL: Investigation. F-yW: Investigation. C-sY: Investigation. S-gS: Investigation. H-cW: Investigation. X-yW: Conceptualization. S-yY: Conceptualization, Methodology.
