# Supplementary material for: Short-term and long-term effectiveness of acupuncture and Tuina on knee osteoarthritis: study protocol for a randomized controlled trial
Source: Front Neurol. 2023 Nov 24;14:1301217. doi: 10.3389/fneur.2023.1301217 (PMC10751577; doi:10.3389/fneur.2023.1301217)
Supplement: Supplementary file 1 [file Data_Sheet_1.docx]

***Supplementary Material***

# Diagnostic criteria

KOA [diagnosis follows the Chinese Orthopaedic Association diagnostic criteria](javascript:;), which is FA01.0 on ICD-11 (1-2).

Diagnosis requires the presence of criterion (1) as well as any other two items. The items contains:

1. recurrent knee pain within the past month;
2. x-ray (standing or weight-bearing position) evidence of narrowing of the asymmetric joint space, osteophyte formations at the joint edge, subchondral bone sclerosis, and/or cystic degeneration;
3. participants aged 50 years or older with no sex limitations;
4. morning stiffness time of no more than 30 minutes;
5. bone crepitus or a feeling of bone friction during physical activity.

**References**

1. Joint Surgery Group, Chinese Orthopaedic Association. Guidelines for the management of osteoarthritis (The 2018 version). Chin J Orthop. 2018;38(12):705-15.
2. Joint Surgery Group, Chinese Orthopaedic Association. Osteoarthritis Group, Orthopaedic Surgeon Branch, Chinese Medical Doctor Association. National Clinical Medical Research Center for Geriatric Diseases. Editorial Department of Chinese Journal of Orthopedics. Chinese guidelines for the management of osteoarthritis (The 2021 version). Chin J Orthop. 2021;41(18):1291-314.

# Locations of acupoints

| **Locations of acupoints for Acupuncture groups** | | |
| --- | --- | --- |
|  | Acupoints | Location |
| Essential acupoints | *Dubi*(ST35) | On the anterior aspect of the knee, in the depression lateral to the patellar ligament |
|  | *Neixiyan*(EX-LE5) | On the anterior aspect of the knee, in the depression medial to the patellar ligament |
|  | *Zusanli*(ST36) | 3 cun directly below ST35, and one finger-breadth lateral to the anterior border of the tibia |
|  | *Yinlingquan*(SP9) | On the tibial aspect of the leg, in the depression between the inferior border of the medial condyle of the tibia and the medial border of the tibia |
|  | *Yanglingquan(GB34)* | On the fibular aspect of the leg, in the depression anterior and distal to the head of the fibula |
|  | *Ashi* point | The point where the patient feels most pain |
| Adjunct acupoints for yangming  meridian syndrome | *Futu* (ST32) | On the anterolateral aspect of the thigh, on the line connecting the lateral end of the base of the patella with the anterior superior iliac spine, 6 cuna superior to the base of the patella |
|  | *Liangqiu* (ST34) | On the anterolateral aspect of the thigh, between the vastus lateralis muscle and the lateral border of the rectus femoris tendon, 2 cun superior to the base of the patella |
|  | *Heding*(EX-LE2) | On the anterior aspect of the thigh, in the depression superior to the base of the patella |
|  | *Fenglong* (ST40) | On the anterolateral aspect of the leg, lateral border of the tibialis anterior muscle, 8 cun superior to the prominence of the lateral malleolus |
| Adjunct acupoints for shaoyang  meridian syndrome | *Waiqiu*(GB36) | On the fibular aspect of the leg, anterior to the fibula, 7 cun proximal to the prominence of the lateral malleolus |
|  | *Xuanzhong*(GB39) | On the fibular aspect of the leg, anterior to the fibula, 3 cun proximal to the prominence of the lateral malleolus |
|  | *Zulinqi*(GB41) | On the dorsum of the foot, distal to the junction of the bases of the fourth and fifth metatarsal bones, in the depression lateral to the fifth extensor digitorum longus tendon |
|  | *Xiyangguan*(GB33) | On the lateral aspect of the knee, in the depression between the biceps femoris tendon and the iliotibial band, posterior and proximal to the lateral epicondyle of the femur |
| Adjunct acupoints for taiyang  meridian syndrome | *Weizhong*(BL40) | On the posterior aspect of the knee, at the midpoint of the popliteal crease |
|  | *Chengshan*(BL57) | On the posterior aspect of the leg, at the connecting point of the calcaneal tendon with the two muscle bellies of the gastrocnemius muscle |
|  | *Kunlun*(BL60) | On the posterolateral aspect of the ankle, in the depression between the prominence of the lateral malleolus and the calcaneal tendon |
| Adjunct acupoints for three-yin  meridian syndrome | *Xuehai*(SP10) | On the anteromedial aspect of the thigh, on the bulge of the vastus medialis muscle, 2 cun superior to the medial end of the base of the patella |
|  | *Yingu*(KI10) | On the posteromedial aspect of the knee, just lateral to the semitendinosus tendon, in the popliteal crease |
|  | *Xiguan*(LR7) | On the tibial aspect of the leg, inferior to the medial condyle of the tibia, 1 cun posterior to SP9 |
|  | *Sanyinjiao*(SP6) | On the tibial aspect of the leg, posterior to the medial border of the tibia, 3 cun superior to the prominence of the medial malleolus |
|  | *Taixi*(KI3) | On the posteromedial aspect of the ankle, in the depression between the prominence of the medial malleolus and the calcaneal tendon |
|  | *Taichong*(LR3) | In the depression anterior to the junction of the first and second metatarsal bones |
|  | *Gongsun*(SP4) | On the medial aspect of the foot, anteroinferior to the base of the first metatarsal bone, at the border between the red and white flesh |

# Detailed movements

## 3.1 Part 1: preparation activity

Adequate warm-up exercise before training for about 5 minutes, mainly including the neck, shoulder, lumbar spine, hip, knee, ankle and other big joint activities, so that the musculoskeletal system to get "warm up".

## 3.2 Part 2: formal training

Advanced criteria (picture of Scoring Standards)【the scores corresponding to laboursome were 5 and 6; very laboursome corresponds to 7, 8, 9 scores; less than 3 scores are advanced】(Table 1)

| **Table 1** | | |
| --- | --- | --- |
| **scale** | **Difficulty Level** | **The feeling of exercise** |
| 0 | Nothing at all | the sensation of sitting or simply standing |
| 1 | Extremely weak |  |
| 2 | Very weak | light |
| 3 | Weak | the sensation of walking or exercising slightly |
| 4 | Moderate |  |
| 5 | Strong | put a little pressure on yourself to complete the movement |
| 6 | Very strong |  |
| 7 | Extremely strong | strongest |
| 8 | Absolute maximum | try your best to complete the movement possibly |

## 3.3 Part 3: ending stage

It mainly stretches the muscles in the thighs and calves and so on.

(To the next page)

| **the first stage** | | |
| --- | --- | --- |
| [objective](javascript:;): For achieving a satisfactory level of exercise intensity, it is necessary to reach a range of 5-6 points. Should one find themselves comfortable when performing a certain exercise, it is recommended to increase the exercise intensity. Inversely, if the exercise is unable to be completed slowly or with proper balance, or if knee pain or swelling persists for greater than one day post-training, it is advised to refrain from increasing the intensity. | | |
| **Name** | **Detail of movement** | **Increase the intensity** |
| Knee extension training | Sitting in a chair with your back and thighs supported, and your feet flat on the floor. Gradually lift your heels until your legs are completely straightened (achieving a full ankle extension). Hold the position with your thighs secured to the chair for approximately 5 seconds before slowly bending your knees and returning your feet to the floor.  It’s imperative to keep your thighs planted firmly on the chair throughout the entire exercise. | As you progress, ankle weights may be added in increments of 1kg each. |
| Chair up training | For improving lower body strength, place a chair against a wall for optimal back support. Begin by leaning slightly forward and bending your knees until they are directly above your toes. Gradually stand up, pausing momentarily, and then slowly lower yourself back into the chair. During this exercise, it is essential to maintain proper knee and toe alignment, steering clear of any inward knee movement. If able, refrain from using your hands for support. | Split position training: Sit up with the training leg slightly closer to the chair and the non-training leg farther away from the chair, placing more power on the working leg to training. |
| Side leg standing training | Standing upright, and holding onto a chair or placing your hand against a wall for support. While keeping your knees straight, slowly lift one leg so that your thigh moves outward alongside your heel. Hold this position for approximately 5 seconds before slowly lowering your leg.  Throughout the exercise, be sure to maintain proper form-standing straight with minimal boddy movement, avoid lifting your leg by rotating your hips or leaning to one side. Keep your knees and toes both pointing forward, and your heels slightly behind your body. | Using an elastic sports band and placing it around both of your ankles of both feet to increase resistance while performing the exercise. |
| Calf bend training | Standing slightly forward and propping your upper limb on a table or high stool. Gradually bend one knee, bringing your heel towards your hip. Once your heel is aligned with your knee and is at a 90-degree angle from your thigh, hold for approximately 5 seconds before slowly lowering your foot back onto the ground. For optimal results, make sure to keep your knees together throughout the entire exercise. | As you progress, ankle weights may be added in increments of 1kg each. |
| Heel raises | Standing with your leg straight and hold onto a chair or place your hand against a wall for added support. Gradually rise onto your toes and raise your heels about 5 seconds before slowly returning to the ground. To ensure proper form, try to avoid swaying back and forth and keep your body upright and still. | Standing on one leg and do one side calf raises alone. Alternatively, gradually add ankle weights in increments of 1kg each. |

| **the second stage** | | |
| --- | --- | --- |
| [objective](javascript:;): To achieve optimal results, it is recommended to aim for an exercise intensity level of 7-8 points. Increase the intensity of the exercise if you comfortable completing it. | | |
| **Name** | **Detail of movement** | **Increase the intensity** |
| Knee extension training | Same as the first stage. | Same as the first stage. |
| Chair up training | Same as the first stage. | Same as the first stage. |
| Stand against the wall  (new movement) | Position yourself sideways against the wall. Lift the leg closest to the wall, bending the hip to a 60-degree angle with the knee below the hip. Simultaneously, bend the knee to a 90-degree angle, ensuring that the heels are below the knees. Maintain slight knee flexion in the standing leg. Apply gentle pressure against the wall with the lifting leg against it, using even force on the thigh, knee, and ankle. Hold this position for 10 seconds before slowly lowering your foot back to the ground. Take a few minutes to rest before continuing.  Throughout the exercise, it is important to keep the hips, knees, and ankles in contact with the wall. | Gradually intensify the workout by increasing 5 seconds when you push against the wall with each workout session. Once you reach the maximum of 30 seconds, do not exceed that time frame to prevent overexertion. |
| Calf bend training | Same as the first stage. | Same as the first stage. |
| One leg heel raises  (new movement) | Supporting yourself by either holding onto a chair or placing your hand on the wall. Standing on one leg, slowly rise onto your toes, lifting your heels off the ground, and hold this position for 5 seconds. Then, gradually lower your heels back to the ground.  Maintain an upright posture throughout the exercise, avoiding any rocking or lateral leaning of your body. | As you progress, ankle weights may be added in increments of 1kg each. |

| **the third stage** | | |
| --- | --- | --- |
| [objective](javascript:;): To achieve optimal results, it is recommended to aim for an exercise intensity level of 7-8 points. Increase the intensity of the exercise if you comfortable completing it. | | |
| **Name** | **Detail of movement** | **Increase the intensity** |
| Knee extension training | Same as the first stage. | Same as the first stage. |
| Chair up training | Same as the first stage. | Same as the first stage. |
| One leg heel raises | Same as the second stage. | Same as the second stage. |
| Crab Dribble(new movement) | Standing face a table, bench, wall, or use your hands for balance if needed. Taking 5 steps sideways in one direction slowly and then 5 steps in the opposite direction. Throughout the exercise, make sure to keep facing forward, leading with your heels as you move laterally. Additionally, keep your toes straight in front and avoid twisting or turning your body or thighs. | Placing an elastic sports band above knees. When your ankles are more than 10cm apart, you should feel the resistance provided by the band. Slightly bend your knees while maintaining tension on the band. |
| Glute bridge  (new movement) | Lying flat on your back with your knees bent. Maintain your feet flat on the mat, approximately 35cm away from your hips. Raise your hips off the ground, ensuring your shoulders remain in contact with the mat. Hold this position for around 5 seconds and slowly lower your hips back down.  During the exercise, it is important to maintain a certain height with your hips and avoid bringing your knees too close together. | For this movement, slightly shift the thigh you wish to train closer to your hips while the other thigh slightly away from your hips. Shift your weight onto the thigh that you want to target (the one closer to your hips). |
| Cycling in the air (new movement) | Lying on your back with your lower back against the floor. Place your hands at your heads, arms open. Lift your legs off the ground and simulate a motion as if riding a bike. While performing the leg drive, exhale and lift your upper body, bringing your right elbow to touch your left knee. Hold for about 2 seconds before returning to the starting position. Then, touch your left elbow to your right knee and slowly return to the starting position. | You can extend the time you hold the airborne position from 2 seconds to 5 seconds. |
